# From CT to Microscopy: Radiological–Histopathological Correlation for Understanding Abdominal Lymphomas

**DOI:** 10.3390/cancers17193264

**Published:** 2025-10-09

**Authors:** Ante Luetić, Martina Luetić, Benjamin Benzon, Danijela Budimir Mršić

**Affiliations:** 1 Department of Diagnostic and Interventional Radiology, University Hospital Split, Spinčićeva 1, 21000 Split, Croatia; aluetic@kbsplit.hr; 2 Department of Pathology, Forensic Medicine and Cytology, University Hospital Split, Spinčićeva 1, 21000 Split, Croatia; mmilat@kbsplit.hr; 3 School of Medicine, University of Split, Šoltanska 2, 21000 Split, Croatia; bbenzon@mefst.hr

**Keywords:** non-Hodgkin lymphoma, CT characteristics, intra-abdominal lymphoma, indolent, aggressive

## Abstract

**Simple Summary:**

A computed tomography (CT) scan is the modality of choice for management of abdominal lymphomas. So far, CT morphological (location/origin and morphological appearance) and functional (post-contrast enhancement) characteristics have not been investigated in correlation with histopathological diagnosis, which was the aim of our study. The study revealed enlarged lymph nodes were a slightly more common CT morphological appearance in the indolent B non-Hodgkin lymphoma (NHL) group, while gastrointestinal (GI) wall thickening and organ and cavity solid masses/infiltrates were more frequent in the aggressive B NHL group. CT postcontrast enhancement showed lymphomas originating from the gastrointestinal tract had the highest enhancement, while histopathological characteristics showed that rare T-cell lymphoma enhancement was more pronounced compared to that of B-cell lymphoma. Our results might help in understanding the biological behavior of lymphomas, which can lead to earlier and more comprehensive diagnosis and treatment.

**Abstract:**

Background: Non-Hodgkin lymphomas (NHLs) are a heterogeneous group of indolent or aggressive lymphoproliferative neoplasms arising from lymph nodes or in extranodal locations. Computed tomography (CT) is the imaging modality of choice, while the definitive diagnosis is confirmed by analyzing tissue samples. The aim of this study was to determine the correlation between CT characteristics and histopathological types of abdominal lymphomas. Methods: A retrospective cross-sectional study included 119 patients with histopathologically confirmed abdominal lymphomas who underwent CT of the abdomen and pelvis prior to treatment. The following CT parameters were extracted: morphological presentation (enlarged lymph nodes/conglomerates, solid mass/masses, gastrointestinal wall thickening, abdominal organ involvement, intra- and extraperitoneal infiltrates), location, two-dimensional size, propagation if present, and postcontrast enhancement. Results: Enlarged lymph nodes were a slightly more common CT morphological appearance in the indolent B NHL group, while gastrointestinal (GI) wall thickening, solid masses, and infiltrates were more frequent in the aggressive B NHL group (*p* = 0.0256). Aggressive B-cell lymphomas had larger size at time of diagnosis compared to other types (*p* = 0.0436). CT postcontrast enhancement showed lymphomas originating from the gastrointestinal tract, which presented as wall thickening, had the highest enhancement (*p* = 0.0065 and *p* = 0.0485). Conclusions: Observed differences in abdominal lymphomas’ histopathological and imaging characteristics including location/origin, CT morphological appearance, and postcontrast enhancement revealed that extranodal lymphomas were more often of the aggressive B-cell type, aggressive B-cell types were larger, and GI tract lymphomas showed the most prominent enhancement. These findings can help in the diagnostic process and enable better management of lymphomas.

## 1. Introduction

Non-Hodgkin lymphomas (NHLs) represent a heterogeneous group of solid lymphoproliferative neoplasms that affect individuals across a broad age range. These malignancies arise from the monoclonal proliferation of precursor B or T lymphocytes [1,2,3]. Over the past decade, the global incidence of NHL has been increasing, making it one of the ten most common cancers worldwide, with approximately 550,000 new cases diagnosed annually. The number of cases is rising, particularly in developed and aging populations, with the highest incidence reported in Australia and New Zealand, North America, and Northern Europe [4,5]. In the latest (fifth) edition of the WHO classification of hematolymphoid neoplasms, NHLs are classified according to their cell of origin within the categories of precursor or mature B-cell and T-cell neoplasms [6]. Based on their clinical behavior and prognosis, NHLs can be divided into indolent and aggressive subtypes [3]. Indolent lymphomas, although initially slow-growing, may progress to more aggressive and potentially fatal forms over time [7]. Follicular lymphoma (FL) is the most prevalent among indolent B-cell neoplasms, alongside other entities such as chronic lymphocytic leukemia/small lymphocytic lymphoma (CLL/SLL) and marginal zone lymphoma (MZL) [6,8]. In contrast, diffuse large B-cell lymphoma (DLBCL), Burkitt lymphoma, and mantle cell lymphoma (MCL) represent aggressive entities [9,10]. T-cell NHLs are relatively rare, accounting for approximately 15% of all NHL cases, and encompass a heterogeneous group of T-cell neoplasms such as peripheral T-cell lymphoma (PTCL) and anaplastic large cell lymphoma (ALCL) [11,12]. Depending on the site of involvement, all aforementioned subtypes can be classified as either nodal lymphomas when arising in lymph nodes or extranodal lymphomas when affecting extranodal lymphatic tissue or organs such as the skin, brain, lungs, gastrointestinal (GI) tract, liver, and spleen [13]. Nodal disease typically presents with lymphadenopathy, whereas extranodal lymphomas manifest with symptoms related to the affected organ [14]. Among extranodal sites, which are less commonly involved than lymph nodes, the gastrointestinal tract represents the most frequently affected location [15].

Computed tomography (CT) is the imaging technique of choice for detection and staging of lymphomas, providing precise information about the location and extent of the tumor [13,16,17]. Nodal lymphomas typically present as enlarged lymph nodes with homogenous enhancement. However, nodal involvement may occasionally exhibit heterogeneous enhancement due to necrosis or cystic change [13]. CT imaging of extranodal lymphomas depends on their location which can be in any organ, but the most common location is the gastrointestinal tract [18,19]. NHLs arising in the stomach, small bowel, colon, and esophagus can have various imaging features including a polypoid mass, ulceration, pseudo-aneurysmal dilatation, fold thickening, or a bulky bowel wall. Rarely their manifestation is bowel stenosis [10,16,20]. On the other hand, the most common pattern of NHL involvement in solid organs such as the liver, spleen, pancreas, kidneys, uterus, and prostate is diffuse infiltration, typically presenting as organomegaly. Other forms of disease presentation in these organs include solitary or multiple intraparenchymal lesions mostly seen in the liver, spleen, and kidneys. NHLs of the adrenal glands and ovaries manifest as bilateral organ enlargement [13,20,21]. All of these masses are quite homogenous and very rarely develop heterogeneous enhancement due to central necrosis. Calcifications are also uncommon and, when observed, indicate a prior treatment [13]. However, many of these imaging features are not specific to lymphomas and can be related to other entities such as infarction, acute or chronic inflammation, and primary and metastatic neoplasms [20,22]. Imaging techniques can narrow the differential diagnosis using other relevant clinical information, but a definitive diagnosis is established by analyzing tissue samples [16,19].

While certain studies have reported subtle variations in imaging characteristics related to the specific lymphoma subtypes, the relationship between CT imaging features and histopathological findings has not been explored [10,19,23]. This subject is of significant medical importance due to the varying clinical approaches and treatment strategies required for different types of lymphoma [24,25]. In this study, we investigated the correlation between CT findings and histopathological diagnosis in cases of intra-abdominal lymphomas, as this region represents the most common site for both nodal and extranodal involvement.

## 2. Materials and Methods

### 2.1. Study Design and Patients

**Figure 1:**
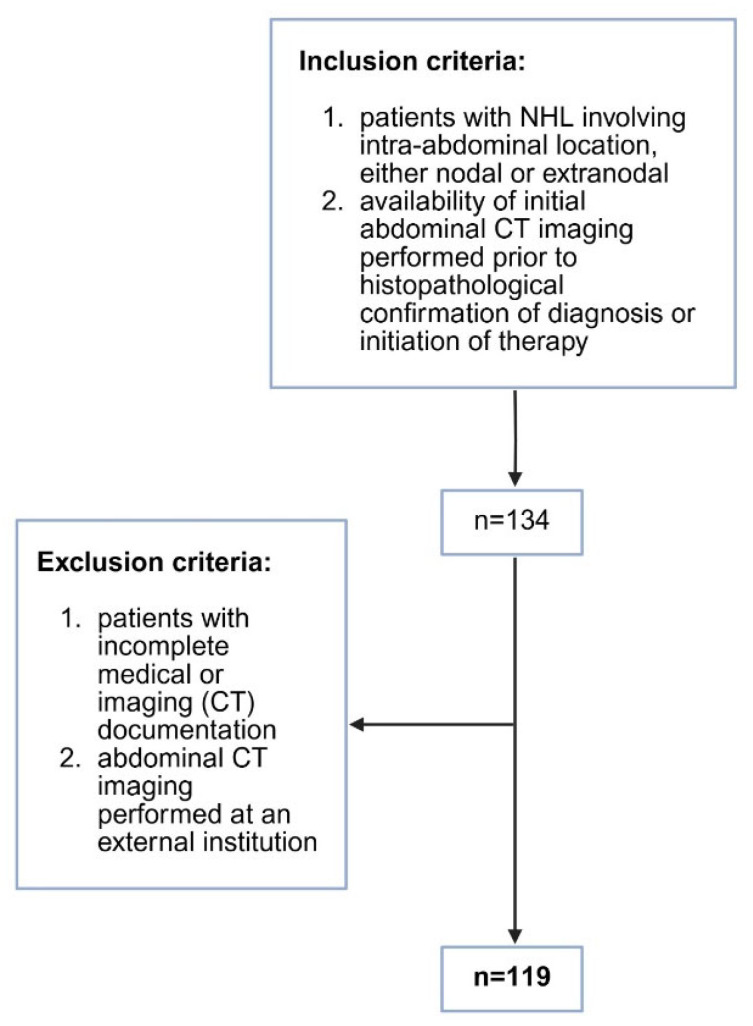
Flow chart of the selected study population.

A retrospective cross-sectional study was conducted at the Department of Diagnostic and Interventional Radiology, University Hospital Split. Patients diagnosed with non-Hodgkin lymphoma in the period between June 2019 and December 2024 were included in the study. Patients’ medical histories were retrieved from the Hospital Information System. Inclusion criteria were as follows: (1) patients with NHL diagnosed in our Institution involving intra-abdominal location of lymphoma, either nodal or extranodal; (2) availability of initial abdominal CT imaging performed prior to the histopathological confirmation of diagnosis or initiation of therapy, which led to a total number of 134 potential patients for the study. After that, we excluded 15 patients based on the exclusion criteria: (1) patients with incomplete medical or imaging (CT) documentation, *n* = 10; (2) initial abdominal CT imaging prior to the treatment performed at an external institution, *n* = 5. The study was approved by the Ethics Committee of University Hospital Split (class: 520-03/25-01/187; registration number: 2181-147/01-06/LJ.Z.-25-02, Chairperson Associate Professor LJ. Z. on 25 June 2025) and was conducted under all ethical principles of the Seventh Revision of the Helsinki Declaration from 2013. Due to the retrospective nature of the study, informed consent was waived. The inclusion and exclusion criteria are shown in Figure 1.

### 2.2. CT Imaging Analysis

**Figure 2:**
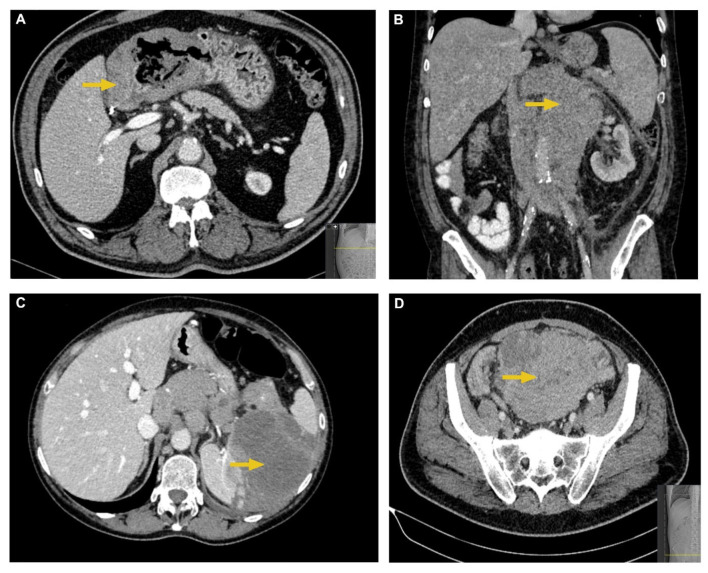
Typical MSCT characteristics of abdominal lymphomas. (**A**) Axial postcontrast CT image shows irregular, circumferential, and hypoattenuating thickening of antral and pyloric gastric wall in a patient with DLBCL. (**B**) Coronal postcontrast CT image shows an extensive, retroperitoneal lymph node conglomerate which encapsulates the aorta and inferior vena cava in a patient with FL. (**C**) Axial postcontrast CT image shows a large hypodense and hypoenhancing splenic mass in a patient with DLBCL. (**D**) Axial postcontrast CT image shows a large, heterogeneous intraperitoneal mass which displaces the bowel loops in a patient with DLBCL.

A 128 multislice CT (MSCT) Siemens Somatom Definition AS, Erlangen, Germany, was used in this study. The imaging acquisition protocol for the abdomen and pelvis included precontrast and postcontrast scanning in the venous phase after intravenous application of low-osmolarity iodine contrast. This protocol is used in our Institution when first diagnosing a suspected lymphoma. CT parameters used were a tube current of 120 to 200 mAs and a tube voltage of 120 kVp, automatically adjusted depending on a patient’s physical characteristics, such as weight. Both precontrast and postcontrast imaging data were analyzed. The following parameters were extracted: morphological presentation (enlarged lymph nodes/conglomerates, organs’ solid mass/masses, gastrointestinal wall thickening, and intra- and extraperitoneal infiltrates/masses), location (specific abdominal or pelvic organ, gastrointestinal tract, intrabdominal or pelvic cavity, intra- or extraperitoneal lymph nodes), two-dimensional size of tumor tissue on axial scans, regional and distal propagation if present, and postcontrast enhancement. Postcontrast enhancement of tumor tissue was measured by measuring CT density in Hounsfield Units (HU) before and after the contrast application. It was measured on axial scans by circling a region of interest (ROI) at the same carefully chosen place before and after the contrast application. All measurements were performed by a radiologist with 10 years’ experience in abdominal imaging interpretation. Examples of different MSCT presentations of abdominal lymphoma are shown in Figure 2.

### 2.3. Histopathological Confirmation of Diagnosis

**Figure 3:**
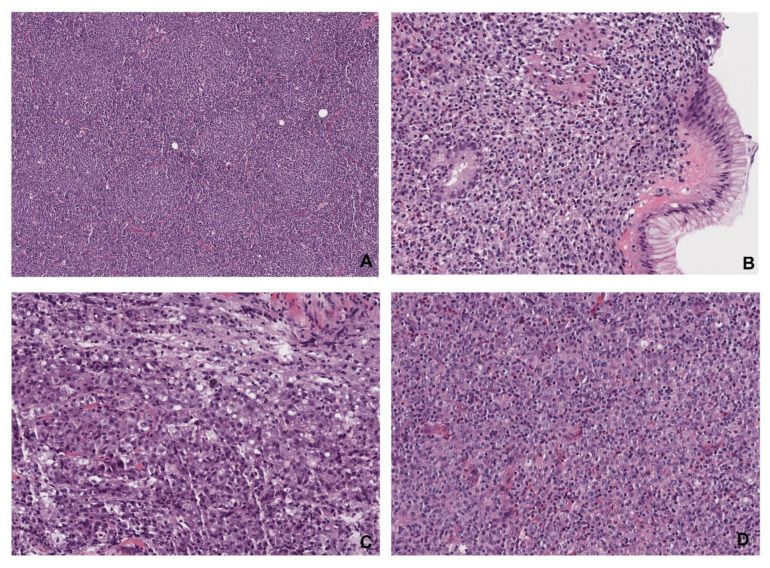
Histopathological images of abdominal lymphomas. (**A**) Follicular lymphoma (magnification ×15) is composed of different-sized follicles containing small-to-medium-sized atypical centrocyte-type lymphocytes. The architecture of a normal lymph node is missing. (**B**) Mucosa-associated lymphoid tissue (MALT) lymphoma (magnification ×60) of the stomach is an indolent extranodal marginal zone lymphoma with diffuse infiltration of lamina propria with infiltration and distortion of glands. (**C**) Diffuse large B-cell lymphoma (DLBCL, magnification ×60) is an aggressive form of lymphoma characterized by diffuse infiltration of large immunoblastic and centroblastic-type lymphocytes. (**D**) Peripheral T-cell lymphoma (PTCL, magnification ×60) is composed of medium-sized-to-large atypical T-cells surrounded by normal lymphocytes, eosinophils, and histocytes.

The final diagnosis of NHLs was established by microscopic analysis of hematoxylin and eosin (H&E)-stained slides prepared from paraffin-embedded tissue samples. Additionally, immunohistochemistry was performed to determine lymphoma subtypes. The most relevant examples of histopathology of abdominal lymphomas are shown in Figure 3.

### 2.4. Statistical Analysis

Before the statistical analyses were performed, three histopathological subgroups were formed, aggressive B NHL, indolent B NHL, and T NHL, as well as four morphological (CT) subgroups: one nodal and three extranodal (gastrointestinal tract, abdominopelvic organ mass/masses, and intrabdominal masses/infiltrates). Categorical data are presented as fractions, while continuous data are presented as either medians with interquartile ranges or arithmetic means and standard deviation. For the analysis of contingency tables, revealing potential difference in lymphoma CT and histopathological subgroups’ distribution differences, Fisher’s exact test or χ^2^ test was used, depending on the sample size. Continuous data, such as the size, morphologic presentation, and contrast enhancement of lymphomas, were modeled with ANOVA or a t test, which is more precisely stated in the text after each analysis. The threshold for statistical significance was set at *p* = 0.05.

## 3. Results

More than 40% of the intra-abdominal lymphomas arose from a lymph node (the nodal subtype, *n* = 49, 41.18%). The other 60% included extranodal lymphomas of gastrointestinal tract origin in a total of 39 patients (32.77%), lymphomas of abdominopelvic organ origin in 18 (15.13%) patients, and lymphomas from intra- and extraperitoneal cavities (the least common) in 13 patients (10.92%), *p* < 0.0001, Table 1.

The gastrointestinal tract lymphomas most commonly arose from the stomach (*n* = 25), followed by the colon (*n* = 9) and the small intestine (*n* = 5). The lymphomas within abdominopelvic organs most commonly involved the spleen (*n* = 7), kidneys (*n* = 5), ovaries (*n* = 2), uterus (*n* = 2), liver (*n* = 1), and pancreas (*n* = 1).

The most frequent histopathological subtype of the nodal lymphoma group was the B-cell type, which encompassed almost the same distribution of aggressive and indolent subtypes of B-cell lymphomas (*n* = 24 (48.98%) vs. 23 (46.94%), respectively), while T-cell lymphomas were found in only 2 patients (4.08%). In contrast, the most frequent histopathological subtype of the extranodal lymphomas was aggressive B NHL in all three extranodal locations, including gastrointestinal tract, abdominopelvic organs and intra- and extraperitoneal cavities (*n* = 25 (64.10%), 9 (50%), and 10 (76.92%), respectively), followed by indolent B NHL and a very small proportion of T-cell lymphomas. However, this distribution was not statistically significant (*p* = 0.4968) (Table 2).

At the time of diagnosis, the majority of the nodal lymphomas had abdominal metastases in other lymph nodes, in comparison to the extranodal lymphomas, roughly two thirds of which had lymph node metastases, *p* = 0.0001 (Table 3). Intraparenchymal metastases were most common in the case of abdominopelvic organ location and least common in the nodal type of lymphoma, *p* = 0.0056. There was no difference in local propagation characteristics of lymphomas, since the majority of nodal and extranodal lymphomas locally propagated into surrounding tissues (*p* = 0.334) (Table 3).

Radiological characteristics on MSCT scans significantly differed depending on the histopathological types and locations of subtypes of abdominal lymphomas. There was a discrepancy between the number of nodal lymphomas based on initial MSCT reporting (*n* = 57) and nodal lymphomas based on subsequent histopathologic confirmation (*n* = 49) due to the fact that some lymph nodes/conglomerates that were described on CT were classified as part of abdominopelvic organs in pathology reports. The majority of nodal types were presented as lymphadenopathy and lymph node conglomerates (*n* = 52, 91.23%), while gastrointestinal tract lymphomas were commonly presented as wall thickening (*n* = 36, 90.00%). In abdominopelvic organs, the solid mass or masses were the only presentation in all cases (*n* = 11, 100%), and intra- and extraperitoneal cavity lymphomas included infiltrates or solid mass at almost the same incidence (*n* = 6, 54.55% infiltrates, *n* = 5, 45.45% masses), *p* < 0.0001 (Table 4).

A significant difference was observed between MSCT presentation and the histopathological type of lymphomas. Intra- and extraperitoneal infiltrates/masses were commonly aggressive B NHL, enlarged lymph nodes/conglomerates were most commonly indolent B NHL, and solid mass/ess were typically aggressive B NHL, as well as GI wall thickening. The least common T type of lymphoma was presented as GI tract wall thickening or enlarged lymph nodes; no T-cell lymphomas presented as an infiltrate or a solid mass, *p* = 0.0264 (Table 5).

**Figure 4:**
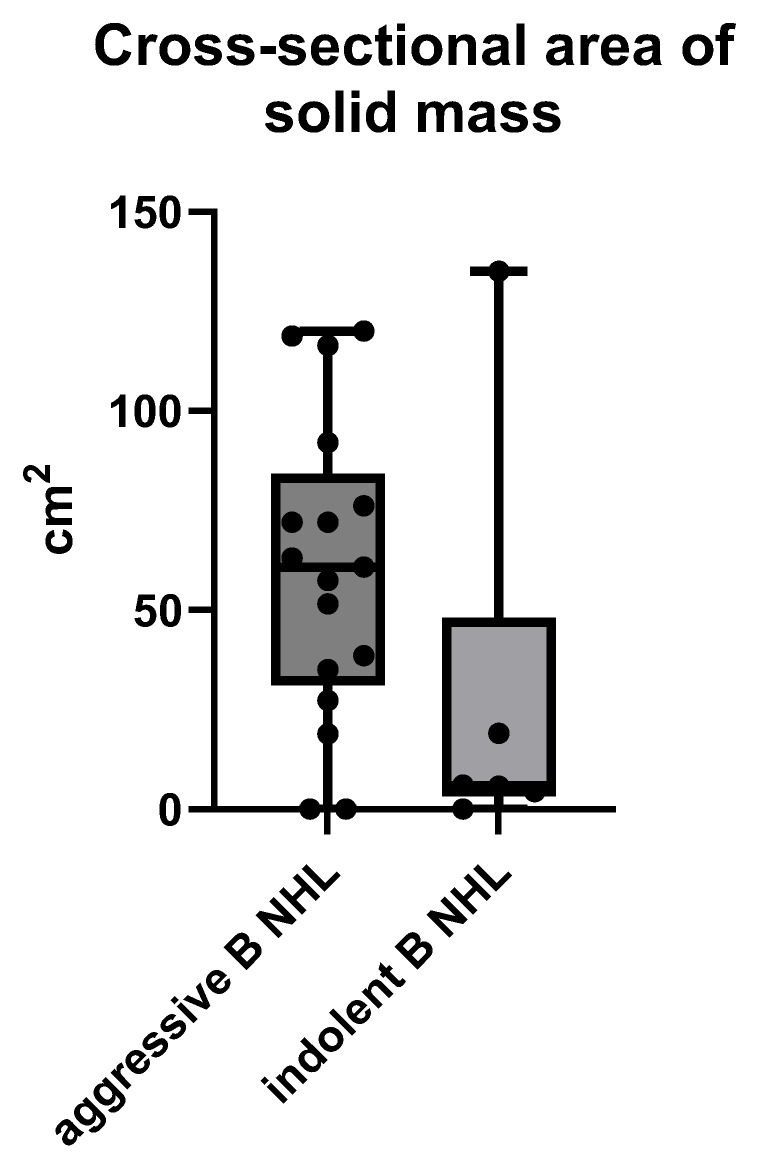
The size of an MSCT solid mass B NHL presented by the largest cross-sectional area in cm^2^ measured on MSCT scans.

Analyses of the size of B NHLs presenting as a solid mass showed aggressive B NHL to be significantly larger at the time of diagnosis, compared to indolent B NHL, with means ± SD of 60.01 ± 37.08 cm^2^ vs. 28.36 ± 52.62 cm^2^ respectively, *p* = 0.0436 (ANOVA) (Figure 4).

**Figure 5:**
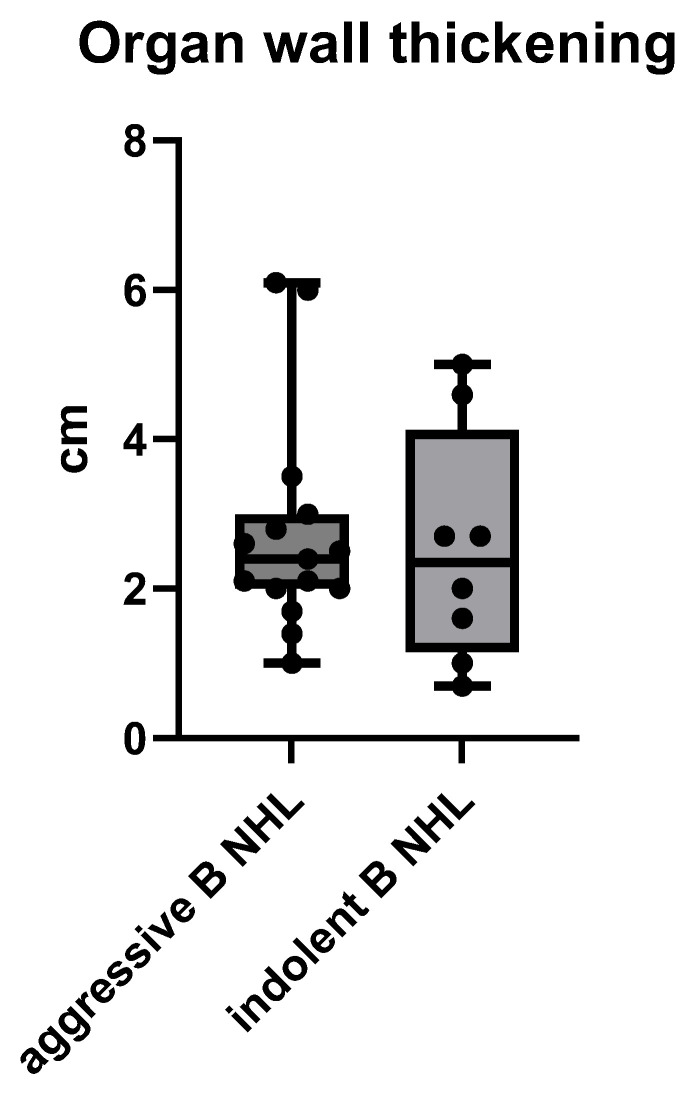
The size of gastrointestinal wall thickening of a B cell NHL presented by its maximum diameter of a wall in cm, measured on MSCT.

The gastrointestinal wall thickening of the B NHL showed no significant difference in the diameter of the wall in both aggressive and indolent B NHL, with means ± SD of 2.74 ± 1.47 cm vs. 2.53 ± 1.57, *p* = 0.7548 (unpaired t test) (Figure 5).

**Figure 6:**
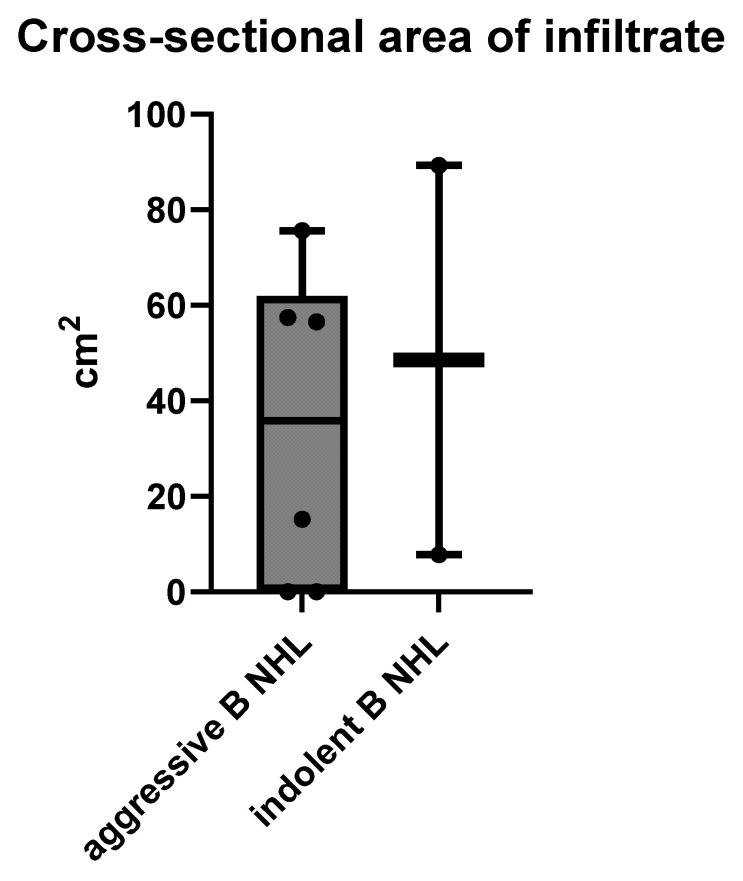
The size of intra-abdominal infiltrates measured on cross-sectional MSCT scans in cm^2^.

Regarding the size of intra-abdominal infiltrates, there was no significant difference between aggressive and indolent B NHL, with means ± SD of 34.13 ± 33.02 cm^2^ vs. 48.52 ± 57.65 cm^2^, respectively (unpaired t test), *p* = 0.6612 (Figure 6).

**Figure 7:**
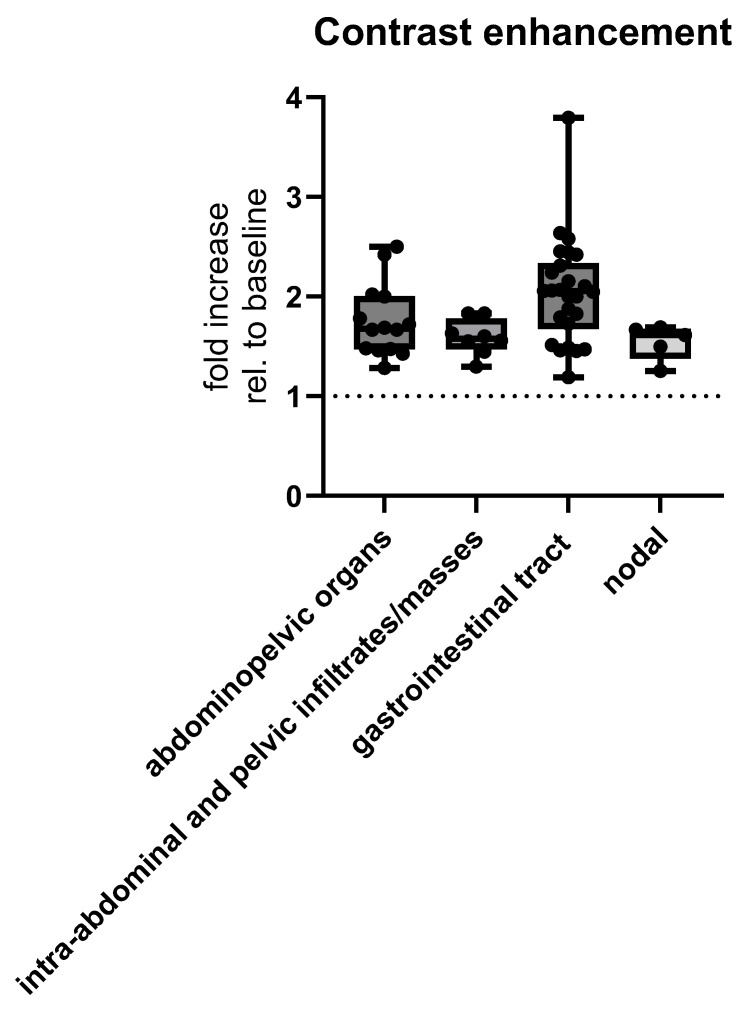
MSCT postcontrast enhancement measured as multiplicative fold increase, i.e., ratio of radiological densities with and without (baseline) contrast, depending on the location of abdominal lymphomas. Abbreviation: rel.—relative.

Contrast enhancement was different depending on lymphoma origin and MSCT presentation. The nodal type had a median multiplicative fold increase of 1.546 ± 0.1781 when compared to CT density without contrast (i.e., baseline), extranodal gastrointestinal tract had a multiplicative fold increase of 2.046 ± 0.5243, intra-abdominal organs had a multiplicative fold increase of 1.757 ± 0.3637, and abdominopelvic infiltrates had a multiplicative fold increase of 1.594 ± 0.1807 (Welch’s ANOVA test), *p* = 0.0065 (Figure 7).

**Figure 8:**
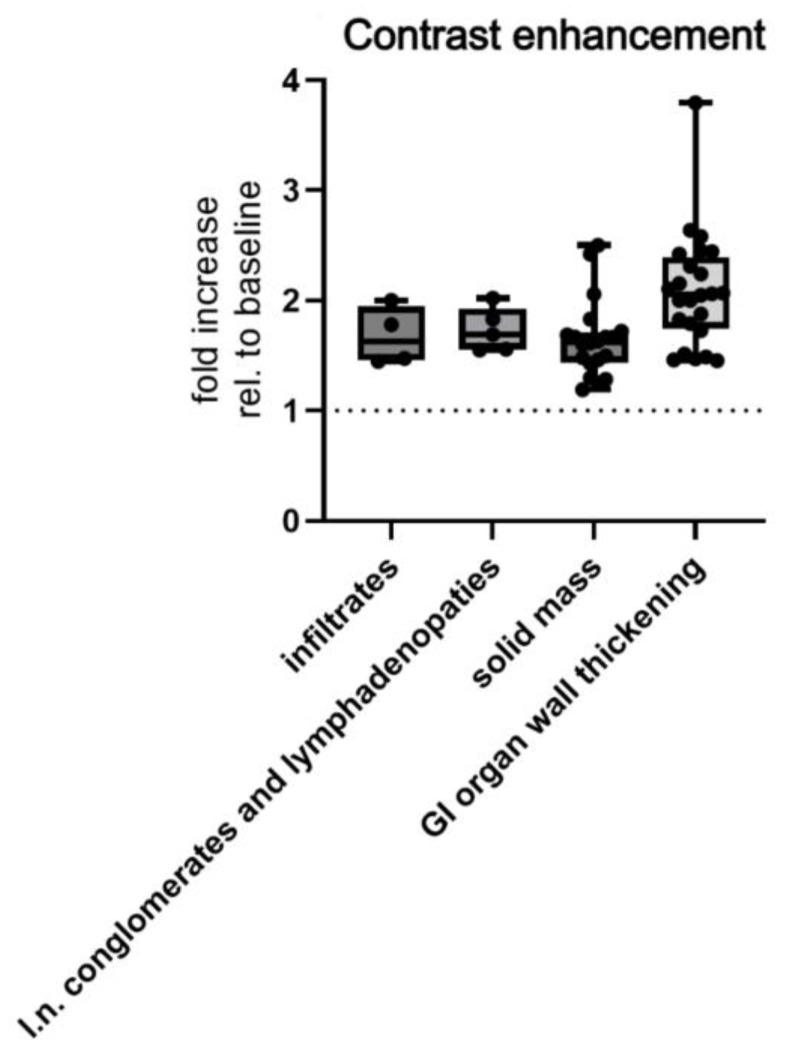
MSCT measured postcontrast enhancement of morphological subtypes of abdominal lymphomas. Abbreviations: rel.—relative; l.n.—lymph node.

Depending on morphological presentation on MSCT, there was also a significant difference in contrast enhancement, which was increased by means ± SD of 2.081 ± 0.5155 HU in GI wall thickening, 1.731 ± 0.1998 HU in enlarged lymph nodes, 1.648 ± 0.3468 HU in solid mass/ess, and 1.675 ± 0.2643 HU in infiltrates (Welch’s ANOVA test, *p* = 0.0485, Figure 8).

**Figure 9:**
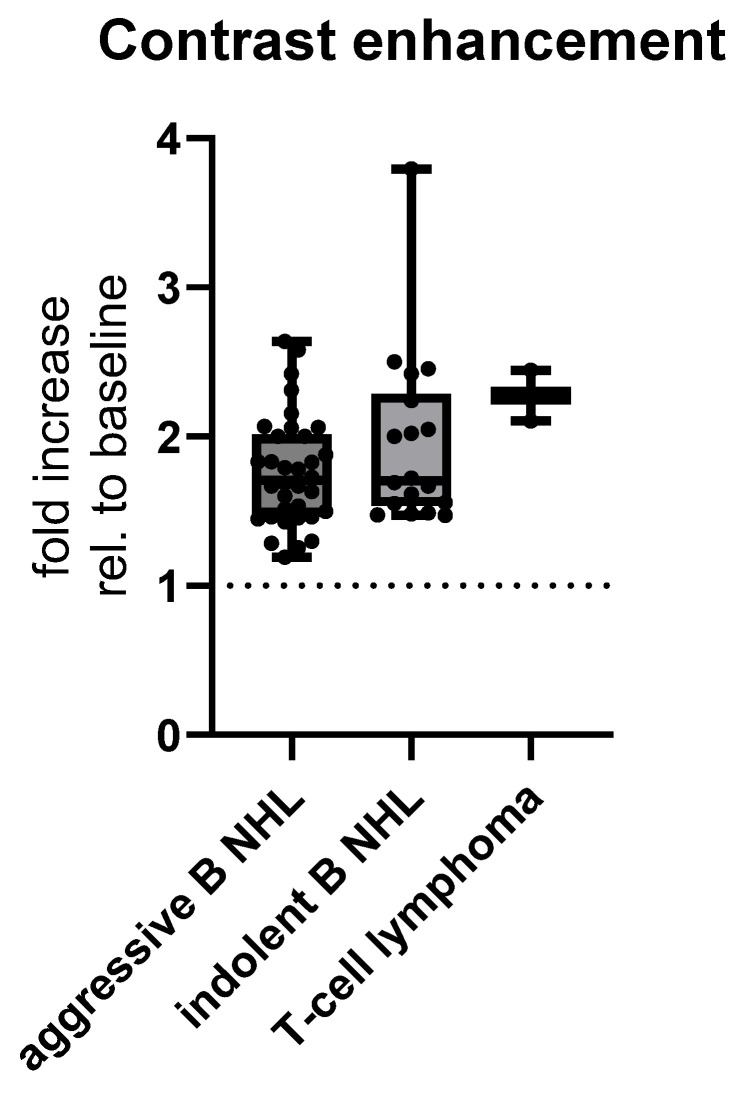
MSCT postcontrast enhancement relative to baseline CT density of histopathological subtypes of abdominal lymphoma. Abbreviation: rel.—relative.

Lastly, contrast enhancement among histopathological subtypes was shown not to be significant among aggressive B NHL, indolent B NHL and T-cell lymphomas, with means ± SD of enhancement of 1.766 ± 0.3694 HU for aggressive B, 1.956 ± 0.5839 HU for indolent B, and 2.274 ± 0.2411 HU for T-cell lymphomas, (test for linear trend, *p* = 0.0561, Figure 9).

## 4. Discussion

The current study revealed that some morphological characteristics on CT scans were associated with histopathological characteristics of abdominal lymphomas. Enlarged regional lymph nodes and conglomerates of lymph nodes were slightly more often due to indolent B-cell NHLs, while GI wall thickening, solid mass/masses and infiltrates were more frequently due to aggressive B-cell NHLs. Postcontrast enhancement of lymphomas further showed significant differences depending on origin and CT characteristics but not histopathological characteristics, revealing that extranodal origin within the GI tract presenting as GI wall thickening showed the strongest enhancement compared to other CT appearances.

In our study, more than 40% of lymphomas arose from lymph nodes (nodal type), and the other 60% were of extranodal origin, most commonly from the GI tract, and less often from organs or the abdominal cavity. This is in accordance with other studies that found slight extranodal predominance with the GI tract being a frequent primary site, similar to our study results [18,26]. Nodal types make up the lower percentage of lymphomas, typically encompass mesenteric, paraaortic, or retroperitoneal lymph nodes, and are often part of a more disseminated disease. While CT presentation of extranodal lymphoma varies, the nodal lymphoma presents with enlarged lymph nodes with regional distribution that can be fused into conglomerates of uniform density and mild enhancement [27]. Of the extranodal lymphomas, GI tract involvement, especially in the stomach, distal ileum, and colon, is characterized by wall thickening, mucosal nodularity, a polypoid or bulky mass, sometimes containing an ulceration, or pseudo-aneurysmal dilatation, and characteristically for all GI lymphomas, there is no significant obstruction of the GI passage [19,28]. Organ involvement includes nodular or mass-like appearance and diffuse organ infiltration, with the spleen, liver, and kidney being the most commonly involved organs [28]. Peritoneal cavity involvement is typically characterized by nodules or masses within the cavity. The extranodal group, due to its heterogeneous morphological appearance, can be misdiagnosed as another (e.g., infective or neoplastic) disease, which delays diagnosis and treatment. In our study, the CT presentations were in accordance with the previous literature; the nodal group presented as enlarged lymph nodes, and extranodal groups were typically presented as wall thickening in the case of GI origin or masses in the case of organ origin.

We further found the nodal lymphomas were typical B-cell NHLs (aggressive and indolent types showed almost the same distribution, although indolent types were slightly more frequent), while extranodal lymphomas were also B-cell NHLs, but aggressive forms predominated over indolent ones. T-cell lymphomas were very rare in our total sample. This is in accordance with other studies which found that B-cell NHLs are overwhelmingly the most common type of lymphoma to affect the abdominal region, with a high percentage of these cases being B-cells in origin [29]. However, the histopathological distribution depends on population and geographical differences, since in Asia and South America, there are slightly more T-cell lymphomas, but despite that they do not predominate over B-cell lymphomas [30]. In our European population, more than 95% of cases were B-cell lymphomas, which is in accordance with previous studies. While nodal lymphomas were slightly more frequently of the indolent B-cell type, all extranodal lymphomas were more often aggressive B-cells. This pattern reflects tendencies in lymphoma behavior and was seen in our study through the contrast enhancement and size of lymphomas. Indolent B-cells typically present with nodal involvement and grow slightly without early symptoms. Aggressive GI or organ lymphomas grow faster, presenting as a mass effect or an organ dysfunction [3].

Furthermore, the MSCT diagnosis of lymphomas is supported by the use of intravenous iodine contrast. Contrast enhancement of the tumor gives information about its vascular characteristics. It is well-known that in the case of lymphomas, the postcontrast enhancement is mild and homogenous [20]. However, the present study revealed it significantly differed depending on lymphomas’ origin and CT presentation, but not histopathological characteristics. Extranodal lymphomas presenting as GI wall thickening demonstrated the most prominent contrast enhancement, in contrast to the mild enhancement of enlarged lymph node presentation. The mucosa and submucosa of the GI wall are highly vascular compared to the vascular component of lymph nodes. Also, the GI tract lymphomas showed the predominance of aggressive B-cell NHLs, which are known for higher cellular turnover, increased metabolic activity, and more profuse angiogenesis, resulting in increased perfusion [31], which may contribute to the better contrast enhancement. In contrast, indolent lymphomas grow more slowly with no prominent angiogenesis. Although we noticed T-cell NHL was enhanced more prominently, it was of no statistical significance. T-cell lymphomas show more aggressive behavior than B-cell lymphomas. This enhancement pattern depends on vascularization/neo-angiogenesis and proliferation activity, which lead to better perfusion and accumulation of iodine contrast in more aggressive forms. However, we think our population sample was lacking in T-cell lymphomas and further studies are needed for stronger evidence.

Besides the relatively small number of T-cell lymphomas in our population sample, other limitations include the cross-sectional design of the study, which cannot explain causal relationships. Also, the lack of follow-up data prevents assessment of clinical outcomes, such as response to therapy or survival. The study was conducted at a single institution, which may limit the generalizability of the findings.

## 5. Conclusions

In conclusion, in the case of abdominal lymphomas, a CT scan is the primary imaging tool and modality of choice for the diagnosis, staging, and follow-up of the disease. This study revealed that some CT morphological (location/origin and morphological appearance) and functional (post-contrast enhancement) characteristics were closely related to histopathological characteristics of abdominal lymphomas. While nodal types of lymphoma were slightly more frequently indolent B-cells, extranodal types were more commonly aggressive B-cells. Enlarged lymph nodes were the most frequent CT presentation for nodal lymphomas, while GI wall thickening, solid masses, and infiltrates represented the most frequent presentation for extranodal types. Aggressive B-cell lymphomas had larger size at time of diagnosis compared to other types. Contrast enhancement is the most pronounced in GI wall thickening on the CT presentation of lymphoma. This can help us to understand the biological behavior of lymphomas, which can in turn improve the diagnostic process overall and enable better management of lymphomas.

## Figures and Tables

**Table 1:** Distribution of intra-abdominal primary lymphomas based on pathology report.

Location	N (%)
Nodal	49 (41.18%)
Extranodal—gastrointestinal tract	39 (32.77%)
Extranodal—abdominopelvic organs	18 (15.13%)
Extranodal—intra-abdominal and pelvic infiltrates/masses	13 (10.92%)
Total (nodal and extranodal)	119 (100%)

Chi-squared test, *p* < 0.0001.

**Table 2:** Distribution of histological subtypes of nodal and extranodal intra-abdominal lymphomas.

N (%)	Aggressive B NHL	Indolent B NHL	T-Cell
**Nodal**	24 (48.98%)	23 (46.94%)	2 (4.08%)
Extranodal—gastrointestinal tract	25 (64.10%)	9 (23.08%)	5 (12.82%)
Extranodal—abdominopelvic organs	9 (50.00%)	7 (38.89%)	2 (11.11%)
Extranodal—intra-abdominal and pelvic infiltrates/masses	10 (76.92%)	3 (23.08%)	0 (0.00%)
**Total extranodal**	44 (62.85%)	19 (27.14%)	7 (17.50%)

Fisher exact test, *p* = 0.4968 (only extranodal subtypes), *p* = 0.1433 (nodal and total extranodal).

**Table 3:** Number of distant lymph node and abdominopelvic organ metastases and local propagation at the time of diagnosis.

**Distant** **Lymph Node** **Metastases** **N (%)**	**Nodal**	**Extranodal—** **Gastrointestinal** **Tract**	**Extranodal—** **Abdominopelvic** **Organs**	**Extranodal—** **Intra-Abdominal and Pelvic** **Infiltrates/Masses**
Positive	47 (95.9)	24 (61.5)	12 (66.7)	9 (69.2)
Negative	2 (4.10)	15 (38.5)	6 (33.3)	4 (30.8)
**Parenchymal** **Metastases** **N (%)**	**Nodal**	**Extranodal—** **Gastrointestinal** **Tract**	**Extranodal—** **Abdominopelvic** **Organs**	**Extranodal—** **Intra-Abdominal and Pelvic** **Infiltrates/Masses**
Positive	8 (16)	9 (23)	10 (56)	6 (46)
Negative	41 (84)	30 (77)	8 (44)	7 (54)
**Local Propagation** **N (%)**	**Nodal**	**Extranodal—** **Gastrointestinal Tract**	**Extranodal—** **Abdominopelvic** **Organs**	**Extranodal—** **Intra-Abdominal and Pelvic** **Infiltrates/Masses**
Positive	47 (96)	32 (82)	14 (78)	13 (100)
Negative	2 (4)	7 (18)	4 (22)	0 (0.00)

Fisher exact test, *p* = 0.0001; Fisher exact test, *p* = 0.0056; Fisher exact test, *p* = 0.334.

**Table 4:** MSCT morphological characteristics of nodal and extranodal abdominal lymphomas.

N (%)	Nodal	Extranodal— Gastrointestinal Tract	Extranodal— Abdominopelvic Organs	Extranodal— Intra-Abdominal and Pelvic Infiltrates/Masses
Infiltrates	1 (1.75)	1 (2.5)	0 (0.00)	6 (54.55)
Lymphadenopathy and conglomerates	52 (91.23)	0 (0.00)	0 (0.00)	0 (0.00)
Solid mass or masses	4 (7.02)	3 (7.5)	11 (100)	5 (45.45)
GI wall thickening	0 (0.00)	36 (90.00)	0 (0.00)	0 (0.00)

Chi-squared test, *p* < 0.0001. Abbreviation: GI—gastrointestinal.

**Table 5:** Histopathological and MSCT characteristics of abdominal lymphomas.

N (%)	Infiltrates	Lymphadenopathy and Conglomerates	Solid Mass or Masses	GI Wall Thickening	Total
**Aggressive B NHL**	6 (75.00)	22 (42.31)	18 (78.26)	22 (61.11)	68
**Indolent B NHL**	2 (25.00)	26 (50.00)	5 (21.74)	9 (25.00)	42
**T-cell**	0 (0.00)	4 (7.69)	0 (0.00)	5 (13.89)	9

Chi-squared test, *p* = 0.0264. Abbreviation: GI—gastrointestinal.

## Data Availability

Data are available upon reasonable request.

## Abbreviations

The following abbreviations are used in this manuscript:

CT	computed tomography
NHL	non-Hodgkin lymphoma
GI	gastrointestinal
WHO	World Health Organization
FL	follicular lymphoma
CLL/SLL	chronic lymphocytic leukemia/small lymphocytic lymphoma
MZL	marginal zone lymphoma
DLBCL	diffuse large B-cell lymphoma
MCL	mantle cell lymphoma
PTCL	peripheral T-cell lymphoma
ALCL	anaplastic large cell lymphoma
MSCT	multislice computed tomography
